# Supplementary material for: Optimized partial freezing protocol enables 10-day storage of rat livers
Source: Sci Rep. 2024 Oct 25;14:25260. doi: 10.1038/s41598-024-76674-6 (PMC11502795; doi:10.1038/s41598-024-76674-6)
Supplement: Supplementary file 1 — Supplementary Material 1 [file 41598_2024_76674_MOESM1_ESM.pdf]

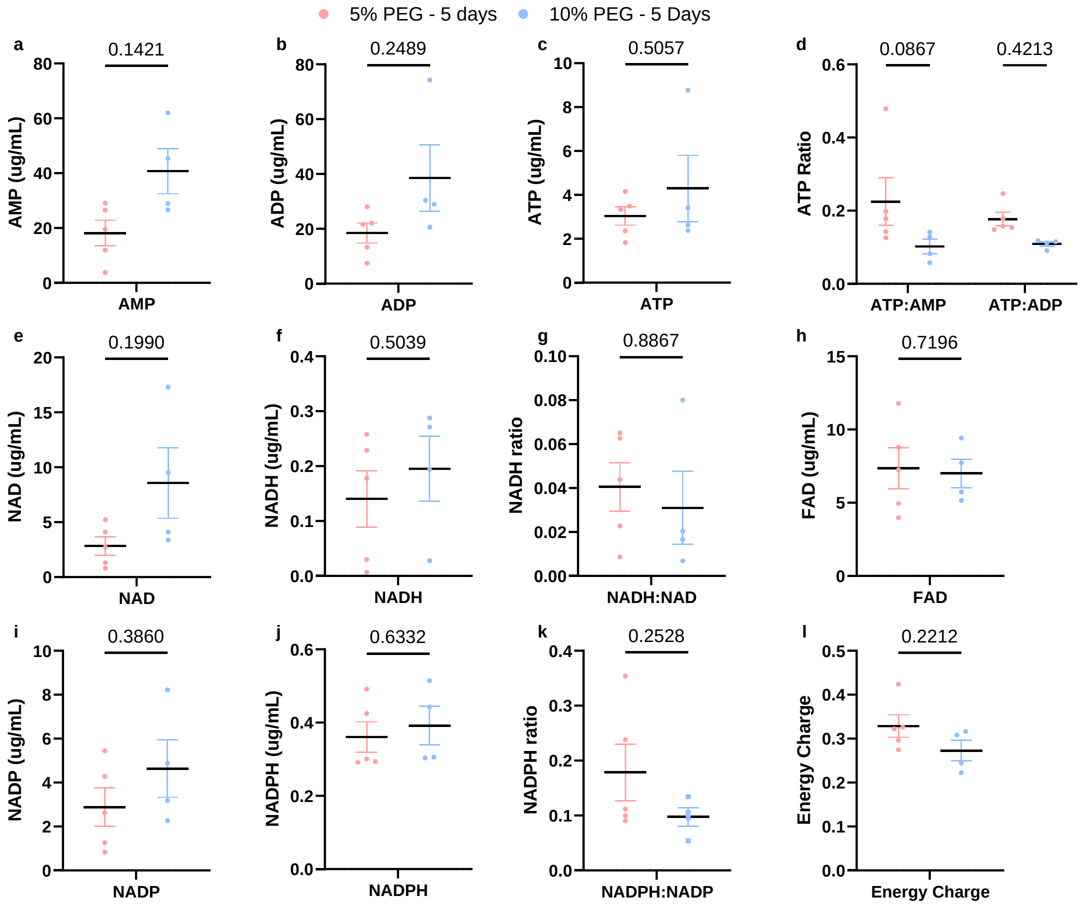

Supplementary Figure 1: Liver Bioenergetics in PF liver Following NMP:

Comparison of bioenergetic molecules between optimized and unoptimized partially frozen livers following NMP showing adenosine monophosphate (AMP) **(A)**, adenosine diphosphate (ADP) **(B)**, adenosine triphosphate (ATP) **(C)**, ATP:AMP and ATP:ADP ratio **(D)**, nicotinamide adenine dinucleotide (oxidized, NAD) **(E)**, nicotinamide adenine dinucleotide (reduced, NADH) **(F)**, NADH:NAD ratio **(G)** flavin-adenine dinucleotide (FAD) **(H)**, nicotinamide adenine dinucleotide phosphate (oxidized, NADP) **(I)**, nicotinamide adenine dinucleotide phosphate (reduced, NADPH) **(J)**, NADPH:NADP ratio **(K)**, energy charge **(L)**.

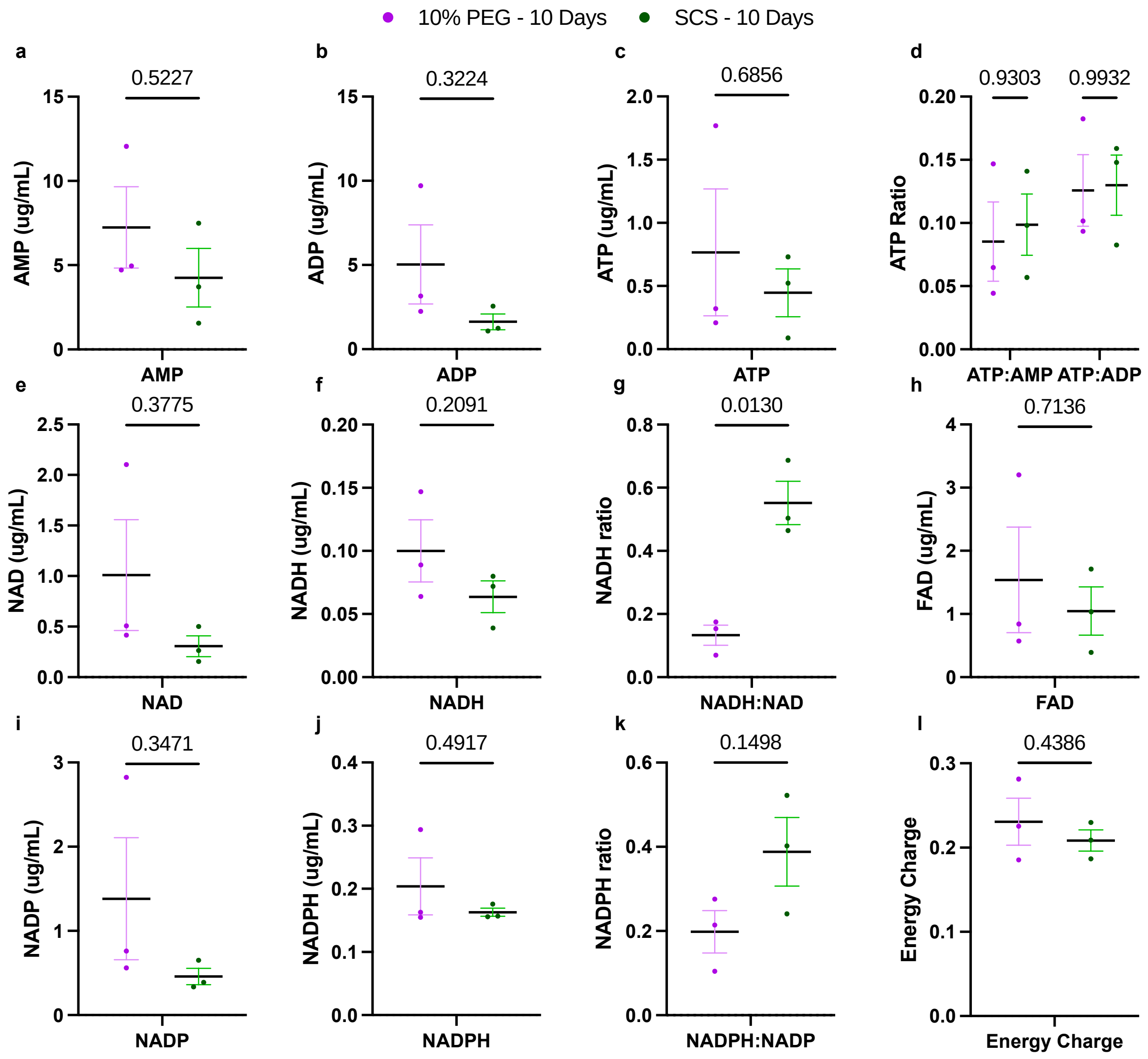

Supplementary Figure 2: Bioenergetic Comparison Between Partial Freezing and Static Cold Storage:

Comparison of bioenergetic molecules between 10-day PF and 10-day SCS livers following simulated transplantation showing AMP **(A)**, (ADP) **(B)**, ATP **(C)**, ATP: AMP and ATP:ADP ratio **(D)**, NAD **(E)**, NADH **(F)**, NADH:NAD ratio **(G)** FAD **(H)**, NADP **(I)**, NDAPH **(J)**, NADPH:NADP ratio **(K)**, energy charge **(L)**.

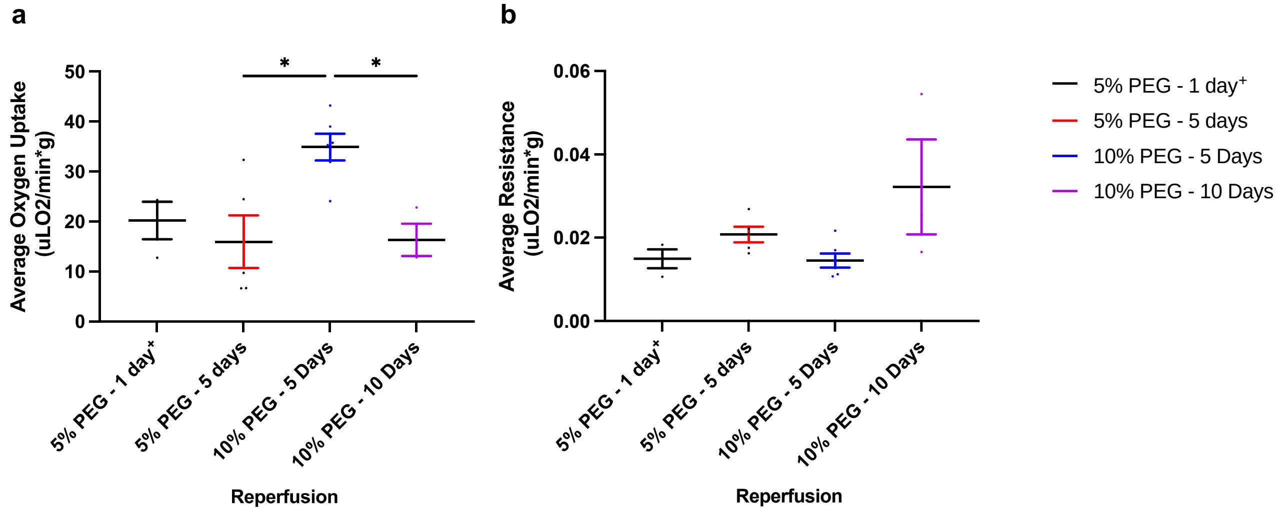

Supplementary Figure 3: Effect of storage duration (1 vs. 5 vs. 10 days) and storage solution (Unoptimized; 5% PEG, Optimized: 10% PEG) on liver function after partial freezing.

(a) The oxygen uptake rate declined from 1-day (black) to 5-day PF (red), but optimizations improved liver function, leading to increased oxygen consumption (blue). However, oxygen uptake decreased after 10 days of storage (purple), indicating a limit to the preservation duration. **(b)** Vascular resistance gradually increased from 1 to 5 days, but solution optimizations led to a decrease in resistance in the time-matched group. However, even with optimizations, a 10-day storage period resulted in a rise in resistance. Stars denote statistical significance: * $p < 0.05$ (two-way ANOVA, followed by Tukey's post-hoc test). Lines: mean, error bars: SEM. $^{+}$*The data originated from Tessier et al.* [25].

5% 10%

Components	Preconditioning (250 mL)	Preloading (250 mL)	Storage (100 mL)	Thawing (250 mL)	Recovery (500 mL)	Blood Solution (100 mL)
Base	250 mL WE	250 mL WE	100 mL UW	250 mL WE	500 mL WE	90 mL WE
Insulin	500 uL	500 uL	50 uL	10 uL	10 uL	1.8 uL
Heparin	2.5 mL	1 mL		1 mL	5 mL	0.9 mL
Hydrocortisone	50 uL	50 uL		50 uL	100 uL	18 uL
Penstrep	1 mL	1 mL		1 mL	2 mL	0.36 mL
L-glutamine	2.5 mL	2.5 mL		2.5 mL	5 mL	0.9 mL
PG		6%	12%	6%		
PEG	2.5 g 5 g	2.5 g 5 g	5 g 10 g	2.5 g 5 g	5 g 10 g	1.8 g
HES		7.5 g		7.5 g		
Raffinose		4.45 g		4.45 g		
Trehalose			1.89 g	4.73 g		
BSA	2.5 g	2.5 g		2.5 g	5 g 37.5 g	6.75 g
3-OMG	4.85 g	4.85 g	1.94 g	4.85 g		
GSH				384 mg	768 mg	138 mg
Snomax			100 mg			
Whole Blood						10 mL

Supplementary Table 1: Solution Formulations:

Table outlining each component in the solutions used during the partial freezing protocol. Components in black are used in the unoptimized solutions, while components in red were changes for the optimized solutions. PG = propylene glycol, PEG = polyethylene glycol 35k, HES = hydroxyethyl starch, BSA = bovine serum albumin, 3-OMG = 3-O-methyl-D-glucopyranose, GSH = L-glutathione reduced.

Components	Supplier	CAS
UW	Bridge to Life	
Williams-E	Sigma-Aldrich	
Insulin	MGH Pharmacy	
Heparin	MGH Pharmacy	
Hydrocortisone	MGH Pharmacy	
Penstrep	Gibco	
L-glutamine	Gibco	
PG	VWR	57-55-6
PEG	Sigma-Aldrich	25322-68-3
HES	AK Scientific	9005-27-0
Raffinose	Chem-Impex	17629-30-0
Trehalose	Sigma-Aldrich	6138-23-4
BSA	Sigma-Aldrich	9048-46-8
3-OMG	Chem-Impex	13224-94-7
GSH	Sigma-Aldrich	70-18-8
Snomax	Snomax	

Supplementary Table 2: Chemical Components:

List of all components used throughout each solution, as well as their respective suppliers.
